# Bochdalek Hernia With Gastric Volvulus in an Adult: Common Symptoms for an Original Diagnosis

**DOI:** 10.1097/MD.0000000000002197

**Published:** 2015-12-28

**Authors:** Mejri Atef, Trigui Emna

**Affiliations:** From the Department of Surgery, Hospital of Jendouba, Jendouba, Tunisia.

## Abstract

Bochdalek hernias in adulthood are rare. Symptomatic Bochdalek hernias in adults are rarer, but may lead to fatal complications. Patients with acute gastric volvulus on diaphragmatic hernia are a diagnostic and therapeutic emergency.

Here, we report a case of a 56-year-old woman diagnosed with epigastric pain, cough, vomiting since 2 weeks and shortness of breath. Complicated Bochdalek hernia was an incidental finding, diagnosed by chest radiograph, computed tomography (CT), and barium swallow study. Stomach was within the thorax in the left side due to left diaphragmatic hernia of a nontraumatic cause.

The patient was prepared for the laparoscopic surgical repair, to close the defect. The patient recovered with accepted general condition and was discharged 9 days later.

Diagnoses of Bochdalek hernias in adulthood are challenging. However, although rare, this possibility should be kept in mind to avoid fatal complications.

## INTRODUCTION

Gastric volvulus is defined as an abnormal rotation of all or part of the stomach around one of its axes. Organoaxial and mesentericoaxial volvulus are distinguished according to the direction of rotation. The most common cause of gastric volvulus is hiatal hernia.^2^ Bochdalek hernia which is the result of a congenital defect in the posterior costal part of the diaphragm in the region of the 10th and 11th ribs can be a cause, but fairly rare, especially in adults.^1^ Most hernias are asymptomatic and found incidentally.^1^ Gastric volvulus is a diagnostic emergency and therapeutic challenge because in acute forms it may lead to gastric strangulation with a high risk of ischemia and necrosis. Gastric volvulus requires surgical treatment, specifically volvulus reduction, reintegration of the stomach into the abdominal and correction of causal factors.^2^ To the best of our knowledge, this is the first report from this region documenting this rare cause of gastric volvulus in adults.

## CASE REPORT

**FIGURE 1:**
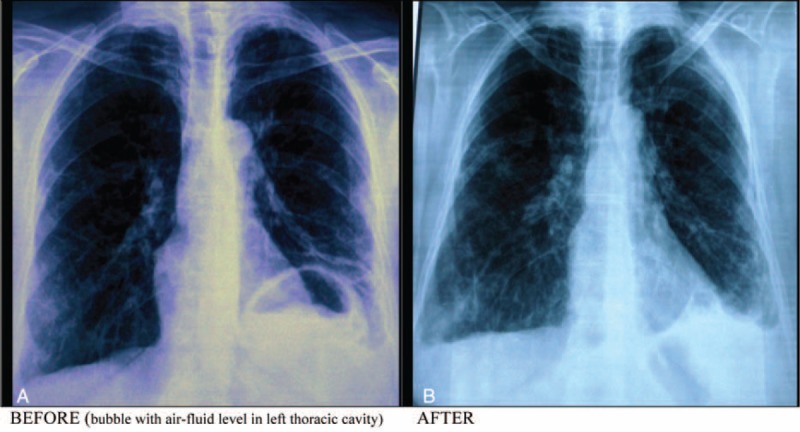
Postero–anterior chest x-ray (before and 1 month after reparation).

**FIGURE 2:**
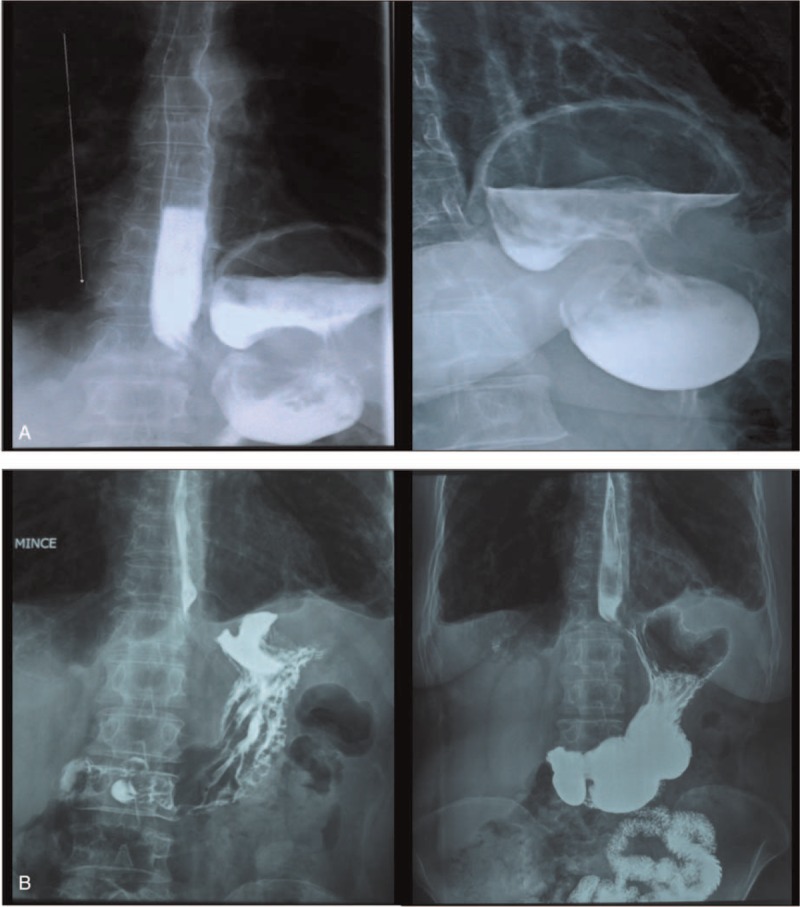
(A) Barium Swallow Study (before reparation). (B) Barium Swallow Study (1 month after reparation).

We have experienced a Tunisian female patient, 56-year-old, not hypertensive, not diabetic, family history and past history were irrelevant. She did not report any reflux symptoms and she denied any use of nonsteroid anti-inflammatory drugs. There was no history of trauma. That patient complained about epigastric pain associated with early satiety and postprandial vomiting for 2 weeks and a moderate form dyspnea. Her physical examination did not reveal any significant abnormality. Laboratory test results showed: white blood cell count of 9090/mm^3^, C-reactive protein (CRP): 73, 64 mg/L, hemoglobin level of 16.3 g/dL, normal cardiac enzymes and blood amylase, lipase, creatinine, and electrolytes were within normal limits. When a nasogastric tube was placed, it could not be advanced into the stomach. A postero–anterior chest x-ray (Figure 1) showed an air bubble with air-fluid level in her left thoracic cavity, and a diaphragmatic hernia was initially suspected. A barium swallow study (Figure 2) confirmed a diagnosis of diaphragmatic hernia with intrathoracic organo-axial gastric volvulus. CT of chest, abdomen, and pelvis with intravenous contrast showed the presence of a left-sided postero-lateral diaphragmatic defect with herniation of the stomach into the thorax.

## METHODS AND EXPLANATION

This case study was carried out in Tunisia. The Ethical Committee, Tunis faculty of Medicine, approved the present study and the patient approved with a written consent prior to the surgery.

It is a birth defect that has remained asymptomatic for 56 years. The diagnosis is made at the stage of acute gastric volvulus on diaphragmatic hernia, which is life threatening. The patient was prepared for laparoscopic diaphragmatic repair.

**FIGURE 3:**
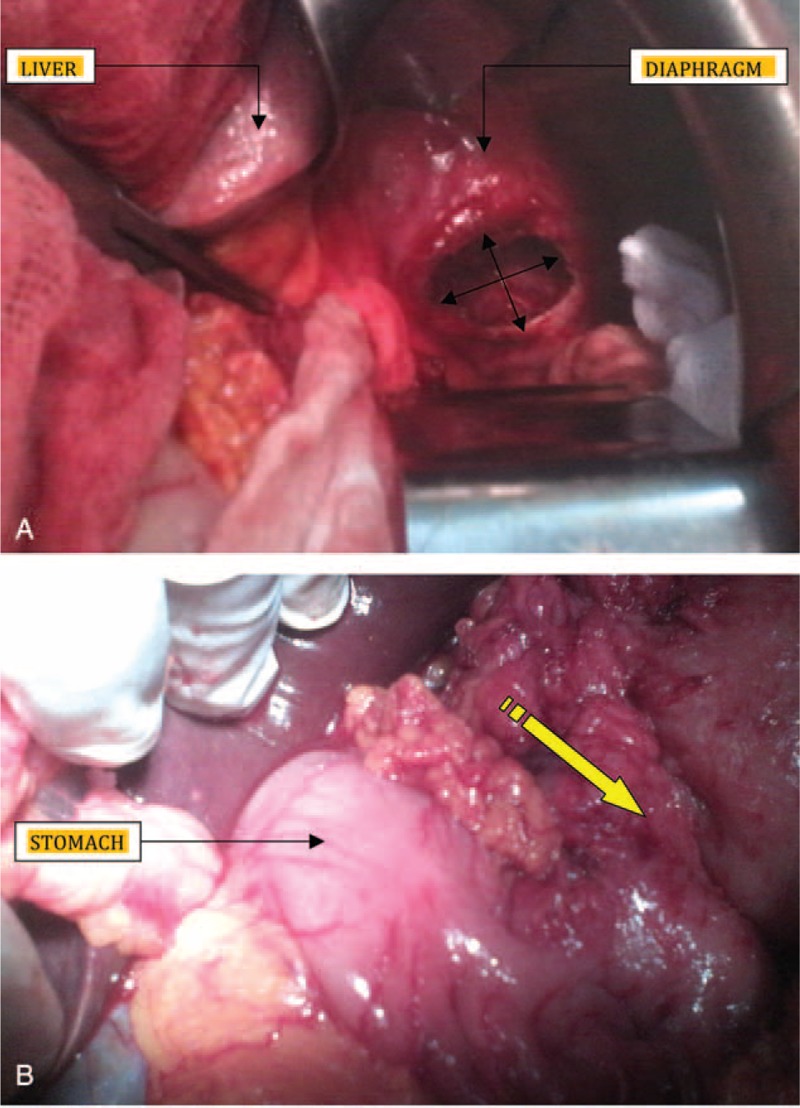
(A) Postero–lateral defect in the left diaphragm. (B) Area of strangulation.

Preoperative assessment included routine investigations. A laparoscopic approach was performed. There was a 10 cm defect (Figure 3) in the left hemi-diaphragm through which the stomach protruded. Dissection of the hernia looks risky there were multiple adhesions. We convert to the laparotomic approach through an upper midline incision. The content was reduced; the stomach was congested but viable (Figure 3). We use 2 to 0 prolene to bridge the diaphragmatic defect. The patient made an uneventful postoperative recovery and was discharged 9 days later.

## DISCUSSION

The foramen of Bochdalek is a 2 to 3 cm opening in the postero lateral aspect of the fetal diaphragm, through which there is communication between the pleural and peritoneal cavities.^3^ This closure failure was first described in 1848 by Bochdalek.^4^ Bochdalek's hernia most commonly manifests during the patient's first few weeks of life. Diagnosis beyond the first 8 weeks of life is estimated to represent 5% to 25% of all Bochdalek's hernias.^5^ In adults Bochdalek hernia is usually asymptomatic.^3^ If symptomatic, the most common presentation is thoracic and abdominal pain, respiratory stress, and bowel obstruction. Presentation with severe symptoms has been reported in 46% of cases, with 32% of mortality because of visceral strangulation and sudden death due to intrathoracic complications.^6^ Few case reports have shown that delayed onset of secondary gastric volvulus associated with congenital diaphragmatic eventration is also possible.^7^ The presence of a diaphragmatic defect may predispose to gastric volvulus because 2 of the 4 ligaments of the stomach (gastrophrenic and gastrosplenic) are connected to the left diaphragm. In patients with congenital diaphragmatic hernia, these ligaments may be elongated or absent.^8^ Borchardt in 1904, described the 3 main clinical signs of gastric volvulus, then named the “Borchardt triad”: unproductive retching, localized epigastric distension, and inability to pass a nasogastric tube.^9,10^ Gastric volvulus is defined as an abnormal rotation of all or part of the stomach around one of its axes. Organoaxial and mesentericoaxial volvulus are distinguished according to the direction of rotation. The most common cause of gastric volvulus is hiatal hernia, but the principal predisposing factor is ligamentous laxity. Gastric volvulus is most often found in the elderly, with a peak incidence around 50.^11^ Regarding gender, it does not seem to be any dominance by most authors.^2^ The diagnosis is suspected when erect chest radiograph images show a high air-fluid level in the chest. Moreover a barium swallow is essential to confirm the diagnosis. Nonetheless, a CT now provides a comprehensive description of the thoracic lesion, including stomach vitality.^2^ A distended, obstructed stomach is prone to ischemia and perforation, which could be fatal. The principles of surgical treatment include detorsion of the volvulus, reduction of the herniated contents, closure of the diaphragmatic defect, and fixation of the stomach to the anterior abdominal wall.

## CONCLUSION

The particular educational message we obtained is to suspect congenital diaphragmatic hernia even in an adult patient. Additionally, surgical treatment is necessary and it can be done with laparoscopic approach.

## References

[1] Yeh-Huang Hung Yu-Hon Chien Sheng-Lei Yan Adult Bochdalek hernia with bowel incarceration. *J Chin Med Assoc* 2008; 71:528–531. 18955188 10.1016/S1726-4901(08)70162-X

[2] Heykal Bedioui Zoubeir Bensafta Volvulus gastrique: diagnostic et prise en charge thérapeutique. *Presse Med* 2008; 37:e67–e76. 17587536 10.1016/j.lpm.2007.03.043

[3] Granier V Coche E Hantson P Intrathoracic caecal perforation presenting as dyspnea. *Case Rep Med* 2010; 2010:1–4. 10.1155/2010/296730 PMC3038627 21331329

[4] Kumar A Maheshwari V Ramakrishnan TS Caecal perforation with faecal peritonitis unusual presentation of Bochdalek hérnia in an adult: a case report and review of literature. *World J Emerg Surg* 2009; 4:16. 19416547 10.1186/1749-7922-4-16 PMC2685771

[5] Nitecki S Bar-Maor JA Late presentation of Bochdalek hernia: our experience and review of the literature. *Isr J Med Sci* 1992; 28:711–714. 1399500

[6] Gedik E Tuncer MC Avci A A review of Morgagni and Bochdalek hérnias in adults. *Folia Morphologica* 2011; 70:1. 21604246

[7] Sinwa PD Gastric mesenteroaxial volvulus with partial eventration of left hemidiaphragm: a rare case report. *Int J Surg Case Rep* 2015; 9:51–53. 25723749 10.1016/j.ijscr.2015.02.034 PMC4392337

[8] Ayala JA Naik-Mathuria B Oluyinka OO Delayed presentation of congenital diaphragmatic hernia manifesting as combined-type acute gastric volvulus: a case report and review of the literature. *J Pediatric Surg* 2008; 43:E35–E39. 10.1016/j.jpedsurg.2007.11.015 18358272

[9] Borchardt M Zur pathologie und therapie des magen volvulus. *Arch Klin Chir* 1904; 74:243–260.

[10] Kotobi H Frederic A Otta E Acute mesenteroaxial gastric volvulus and congenital diaphragmatic hernia. *Pediatr Surg Int* 2005; 21:674–676. 16007427 10.1007/s00383-005-1437-2

[11] Wu MH Chang YC Wu CH Acute gastric volvulus: a rare but real surgical emergency. *Am J Emerg Med* 2010; 28:118e5–118e7. 20006232 10.1016/j.ajem.2009.04.031

